# Miscellanies

**Published:** 1838-04-01

**Authors:** 


### Miscellanies.

### LEEDS PETITION TO PARLIAMENT.

The Members of the LEEDS MEDICAL SOCIETY, consisting of legally-authorized Practitioners, having some time ago had their attention directed to the unprecedented and increasing extent of Quackery, appointed a committee to inquire what means could be adopted for its suppression. On receiving the report of the committee, and at their suggestion, the Society have unanimously determined to Petition the Legislature for that protection against illegal competition to which an honourable Profession is entitled.

It is a fact that in the Borough of Leeds the number of Prescribing Druggists and other illegal practitioners *considerably exceeds* that of the regular Members of the Profession; and the same calculation is applicable to many other places. The uneducated Druggists are now in the full exercise and enjoyment of all those privileges which were intended, by the Act of 1815, to be secured to the Apothecaries; while the latter, after years of preparatory study and expense, have the mortification of seeing *their* harvest reaped by a set of illiterate pretenders.

The following Petition, it is presumed, will point out to the Legislature a sufficient remedy for the evils in question; and the Leeds Practitioners earnestly exhort their brethren throughout the kingdom to second their efforts, by *immediately* sending up to Parliament similar Petitions, signed by all the legally-authorized Practitioners resident in the district where the Petitions originate.

As this is a question touching neither sect nor party, it is hoped that all parties will unite in the endeavour to obtain justice, and that no time will be lost in urging upon the Legislature the necessity of removing this oppressive wrong.

GEORGE WILSON,

Leeds, Jan. 1, 1838. Sec. to the Com.

To THE HONOURABLE THE COMMONS OF THE UNITED KINGDOM OF GREAT BRITAIN AND IRELAND IN PARLIAMENT ASSEMBLED,

This *Petition of the Legally-Authorized Practitioners of Medicine, residing in the Borough of Leeds,*

Humbly Sheweth,

That a very considerable number of Individuals are now practising the different branches of Medicine in England and Wales, without having undergone any preparatory education, or examination by any legally-constituted authority.

That, in consequence of their ignorant administration of remedies, very serious injury is done to the Public Health, and many cases have occurred in which their practices have been undoubted causes of death. Not a few such cases have been already before the public, as matters for legal investigation.

That the extent of employment which such parties are enabled to obtain among the uneducated classes (by advertising false cases, and various other unworthy means) injures materially the practice of regular Members of the Profession; and is more especially oppressive to the Younger Practitioners, who generally depend for their subsistence upon the very classes who are misled by the false statements of pretenders.

That your Petitioners are required by law to undergo an expensive *preliminary* education; and then to engage for several years in severe and unhealthy studies,—subsequently to all which, they are subjected to rigorous examinations by the constituted authorities. Yet the only protection afforded them is by the Apothecaries' Act; which, however, is totally inadequate to redress their grievances, on account of the difficulty of obtaining the required evidenee of illegal practice—the great length of the legal process—and the heavy amount of costs attending prosecutions.

That it is therefore inexpedient and useless to waste further efforts towards the conviction of illegal Practitioners, under the very insufficient powers afforded by the Apothecaries' Act.

That your Petitioners, therefore, beg most respectfully to suggest, as the only means of remedying the above evils, and protecting the public from the dangers of ignorant and empirical practice, that a Clause be added to the Apothecaries' Act, empowering Magistrates to summon before them any party charged with illegally prescribing or practising any branch of Medicine in England or Wales, and in the event of such party failing to produce the Licence of the Apothecaries' Society of London, (or proof of having obtained such Licence) to inflict such a penalty as may seem adequate to prevent a repetition of the offence.

And your Petitioners, &c.

---

### THE LATE MR. TWINING, OF BENGAL.

THE death of this distinguished surgeon, caused a considerable sensation in Calcutta, and throughout India generally, on account of the high character he held amongst his brethren and the public at large. The disease of which he died was obscure, and it is not a little remarkable that he who had so diligently prosecuted pathology, should have expressly requested that his body should not be examined after death! This was a weakness which we should not have expected in such a man as Mr. Twining. It may be observed that for some time before his death, Mr. Twining thought he laboured under angina pectoris. On the 19th of August, 1835, his coachman ran the carriage against a gentleman's buggy, by which accident the gentleman was thrown out, and had his thigh broken. Mr. T. jumped out to help the sufferer, and felt something snap in his own chest. He became instantly sick and faint, followed by a sense of suffocation. The pulse was small, quick, and fluttering. He nearly expired on his way home. The sense of suffocation prevented him from sleeping that night. After a purgative next morning (August 11) he was more comfortable, and slept a little, from which he was awakened by a tickling cough. Some bloody expectoration was thrown up during this and the next day. He slept a little in the night of the 11th, and he took some calomel and blue pill every four hours—and applied sixteen leeches to the hypochondrium, which eased his breathing. He observed that some portion of one lung resembled red-currant jelly. He went out in his carriage on the 22nd and again on the 23rd to see a patient. In the evening of the 22nd he was delirious and very ill. Hitherto he had prescribed for himself. He now consulted Mr. Egerton, who found him with a pulse that could not be counted, but calm and collected. Four grains of calomel and six of pulv. Jacobi—blister to the scrobiculis cordis. He soon afterwards determined in his own mind that his disease was obscure intermittent, and took several doses of quinine. This was followed by considerable amelioration of the symptoms, and elevation of spirits. His pulse, however, continued at 216, with hurried respiration. He slept five hours that night. Next day (25th) he took a purgative, with some relief; but a decided change for the worse took place. He suffered frightfully from difficulty of breathing—and he expired at four o'clock.

The prohibition of a post mortem examination left the real nature of the disease in some doubt, though we think there can be no doubt that an aneurism had burst and pressed upon the large bronchial tubes. The prohibition alluded to, is most extraordinary, and with every disposition to adhere to the ancient but not very wise maxim—" de mortuis nil nisi bonum," we must say, that the medical man who examines the bodies of those who die in the wards of his hospital, and then binds his friends to refrain from ascertaining the nature of an obscure and fatal disease in his own body, is not merely *weak*, but we had almost said *dishonest*. He breaks one of the fundamental and most important commands of his Redeemer—" do unto others as you would they should do unto you." The deceased did an injury to the whole profession. What answer could a medical man expect from the friends of a departed relation, if a *post mortem* were requested? They might well quote the dying injunction of Mr. Twining! Let this be a warning to others!

---

